# Gene expression profile and molecular pathway datasets resulting from benzo(a)pyrene exposure in the liver and testis of adult tilapia

**DOI:** 10.1016/j.dib.2018.08.206

**Published:** 2018-09-05

**Authors:** Reyna Cristina Colli-Dula, Xiefan Fang, David Moraga-Amador, Nacira Albornoz-Abud, Roberto Zamora-Bustillos, Ana Conesa, Omar Zapata-Perez, Diego Moreno, Emanuel Hernandez-Nuñez

**Affiliations:** a CONACYT, Mexico; b Departamento de Recursos del Mar, Cinvestav Unidad Mérida, Mérida, Yucatán 97310, Mexico; c Department of Pediatrics, University of Florida, Gainesville, FL 32610, USA; d ICBR, University of Florida, Gainesville, FL 32610, USA; e TecNM/Instituto Tecnológico de Conkal. Laboratorio de Genética Molecular, Conkal, Yucatán 97345, Mexico; f Centro de Investigacion Principe Felipe, 46012 Valencia, Spain; g Microbiology and Cell Science, Institute for Food and Agricultural Sciences, Genetics Institute, University of Florida, Gainesville, FL 32603, USA; h Universidad Autónoma de Yucatán. Facultad de Ingeniería Ambiental, Mérida, Yucatán 97150, Mexico

## Abstract

Benzo(a)pyrene (BaP), the prototype of polycyclic aromatic hydrocarbons, is known to exhibits genotoxic and carcinogenic effects promoting molecular impacts. The dataset presented here is associated with the research article paper entitled “Transcriptome Analysis Reveals Novel Insights Into the Response of Low-dose Benzo(a)pyrene Exposure in Male Tilapia”. In this article, we presented a transcriptomic characterization of male tilapia exposure to BaP in the short term. This data provides an extended analysis of changes in the gene expression and identification of pathways in the liver and testis of male tilapia exposure to BaP. We used gene set enrichment analysis (GSEA) and sub-network enrichment analysis (SNEA) to identify gene networks and pathways associated with molecular adverse effects of BaP exposure. The data indicates that target pathways related to promoting carcinogenesis such as DNA repair and DNA replication were affected as well as other crucial biological processes. Moreover, to determine whether some of the key reported genes of DNA damage are affected by BaP exposure, Quantitative PCR (qPCR) was performed. Gene set categories and sub-networks are provided and the corresponding signature differences from BaP exposure are listed. The information in these datasets may contribute to understanding the potential carcinogenesis mechanism of action from low BaP exposure.

**Specifications table**

**Table:** 

Subject area	Biology
More specific subject area	Transcriptomics
Type of data	Table, text file.
How data was acquired	mRNA data from RNA-Sequencing (RNA-Seq) technology and bioinformatic analysis were used to identify a molecular signature and pathways affected by BaP exposure.
Data format	Filtered and analyzed.
Experimental factors	Analysis of gene expression profile RNA-Seq data from a BaP experiment
Experimental features	RNA-Seq reads were trimmed and the clean reads analyzed.
	Gene read mapping, differential expression analysis (DE) and GSEA and SNEA were performed from tilapia liver and testis tissue after BaP-treatment.
	Quantitative PCR (qPCR) was used to evaluate the transcriptomic changes observed in BaP-exposed male tilapia.
Data source location	Sample source and tissue harvest were located at Cinvestav-Merida, Yucatan, Mexico. The BaP experiment was carried out at Cinvestav-Merida. Sample analysis was performed at Cinvestav-Merida and the University of Florida (Gainesville, FL, USA).
Data accessibility	Transcriptomic data are presented in this article. All RNA-Seq data were submitted to NCBI׳s Gene Expression Omnibus (GEO) and can be accessed via https://www.ncbi.nlm.nih.gov/geo/query/acc.cgi?acc=GSE116687
	(accession number GSE116687).
Related research article	[1] COLLI-DULA, R. C. et al. Transcriptome Analysis Reveals Novel Insights Into the Response of Low-dose Benzo(a)pyrene Exposure in Male Tilapia. **Aquatic Toxicology**, v. 201, n. 15, pp. 162–173, Aug 2018. ISSN 0166-445×.

**Value of data**

• The data explores the biological mechanism of action of BaP in the liver and testis of male tilapia by a high throughput transcriptomic approach (RNA-Sequencing).
• The data provides ample information of changes in gene expression, subnetworks and functional enrichment analysis associated with several biological processes after BaP treatment.
• The molecular signature identified for BaP exposure is very useful to other researchers that may explore the mechanism of action of BaP in non-model organism such as tilapia.
• New gene sets associated with molecular adverse effects of BaP can be useful in understanding the role of BaP into the activation of apoptotic signals in tilapia.
• This data may contribute to understanding the BaP mechanisms associated with adverse effects in tilapia.

## 1 Data

These data sets provide information on the BaP molecular effects in tilapia testes and liver. Table 1 presents all the primer sets used for qPCR analysis. All primers used were previously validated as indicated in the 2^−ΔΔCT^ method [2]. Of these genes evaluated, *Cyp1b1*, *Ddit4, Gadd45b* and *Fasn* showed significant changes in their levels of expression from BaP exposure (*p *< 0.05) (Table 5 in [1]). Table 2 presents a partial list of the characterization of gene expression profiling of RNA-data by BaP exposure. This data shows a larger number of altered genes in the liver related with adverse molecular effects on the cell cycle and with several other biological processes. Table 3 shows GO categories and Table 4 identifies gene networks altered by a low concentration of BaP in the liver and testis of male tilapia. All significantly altered genes are listed in Table SI as well as identified GO categories and subnetworks which are present.

**Table 1:** Sequences of primers used in the qRT-PCR analysis.

**Gene symbols**	**Description**	**Forward primer 5′→3′**	**Reverse primer 5′→3′**	**Amplicon size (bp)**	**Aligned temp (°C)**
CYP1B1	Cytochrome P450, family 1, subfamily B, polypeptide 1	gggctacaccgtaccaaaga	agcgctctgggtcaaagata	104	56
GADD45	Growth arrest and DNA damage inducible beta	ggagacggtgagtcaagctc	gcggactcgtagactccaac	84	58
DDIT4	DNA-damage-inducible transcript 4	tctcattgacctctgcgttg	accagagcgagctgaaatgt	99	58
IGF2	Insulin-like growth factor 2	gcccttccctttgacattat	gcgccttgtccttgttttag	92	58
TET3	Tet methylcytosine dioxygenase 3	aaaggacccttgtgtcatgc	gtttgcttttcaggcagtcc	80	58
FASN	Fatty acid synthase	gagacggactgccttacagc	gctgcagtctgtggatcaaa	79	58
RPL8	Ribosomal Protein L8	gttgctggaggtggacgtat	ggatgctcaacagggttcat	125	56

**Table 2:** Partial list of the characterization of gene expression profiling of RNA-data. Identified transcripts are involved with electron transport/ATP synthesis, DNA methylation, growth and development, cell cycle machinery and apoptotic signals.

	**Characterization of gene expression profiling of tilapia RNA-seq data**				
			**Liver**		**Testis**
**HGNC symbol**	**Description**	**Fold change**	***p*-value**	**Fold change**	***p*-value**
**Electron transport/ATP synthesis**					
ACLY	ATP citrate lyase a [Source:ZFIN;Acc:ZDB-GENE-031113-1]	−5.5	6.35E−07	−1.0	NS
ATP6V1E1A	ATPase, H+ transporting, lysosomal, V1 subunit E1a [Source:ZFIN;Acc:ZDB-GENE-041212-51]	3.8	2.6E−06		NS
ATAD2	ATPase family, AAA domain containing 2 [Source:HGNC Symbol;Acc:HGNC:30123]	−4.1	2.2E−03	1.1	NS
ATAD2	ATPase family, AAA domain containing 2 [Source:ZFIN;Acc:ZDB-GENE-030131-7003]	−2.1	1.2E−02	1.0	NS
PSMD1	proteasome 26S subunit, non-ATPase 1 [Source:ZFIN;Acc:ZDB-GENE-040426-810]	−1.6	2.1E−02	1.0	NS
ABCE1	ATP-binding cassette, sub-family E (OABP), member 1 [Source:ZFIN;Acc:ZDB-GENE-040426-1995]	1.8	2.5E−02	−1.1	NS
ABCA3	ATP-binding cassette, sub-family A (ABC1), member 3b [Source:ZFIN;Acc:ZDB-GENE-050517-2]	−1.7	2.6E−02	−1.1	NS
CFTR	cystic fibrosis transmembrane conductance regulator (ATP-binding cassette sub-family C, member 7) [Source:ZFIN;Acc:ZDB-GENE-050517-20]	2.2	3.1E−02	−1.0	NS
LIG1	ligase I, DNA, ATP-dependent [Source:ZFIN;Acc:ZDB-GENE-110404-2]	2.0	3.7E−02	1.1	NS
NKAIN1	Na+/K+ transporting ATPase interacting 1 [Source:ZFIN;Acc:ZDB-GENE-040426-1472]	2.1	4.1E−02	−1.3	NS
TAP2	ATP-binding cassette, sub-family B (MDR/TAP), member 3 like 1 [Source:ZFIN;Acc:ZDB-GENE-030616-245]	−2.9	4.5E−02	1.4	NS
AGTPBP1	ATP/GTP binding protein 1 [Source:ZFIN;Acc:ZDB-GENE-081104-267]	−1.7	4.6E−02	−1.0	NS
OPLAH	5-oxoprolinase (ATP-hydrolysing) [Source:ZFIN;Acc:ZDB-GENE-121214-293]	1.6	4.6E−02	1.0	NS
ATP6V1C1	ATPase, H+ transporting, lysosomal, V1 subunit C1b [Source:ZFIN;Acc:ZDB-GENE-041010-104]	−1.6	3.1E−02	−1.2	NS
ATP5I	ATP synthase, H+ transporting, mitochondrial Fo complex, subunit Ea [Source:ZFIN;Acc:ZDB-GENE-070928-12]	−2	1.6E−02	−1.2	NS
ATP7A	ATPase, Cu++ transporting, alpha polypeptide [Source:ZFIN;Acc:ZDB-GENE-060825-45]	−2.2	1.8E−03	−1.1	NS
***Solute carrier families***					
SLC22A7	solute carrier family 22 member 7 [Source:HGNC Symbol;Acc:HGNC:10971]	5.6	2.0E−04	−38.9	5.0E−03
SLC22A7	solute carrier family 22 member 7 [Source:HGNC Symbol;Acc:HGNC:10971]	5.6	2.2E−04	−38.9	4.83E−03
SLC13A1	solute carrier family 13 (sodium/sulphate symporters), member 1 [Source:ZFIN;Acc:ZDB-GENE-031222-3]	5.4	4.2E−02	−1.3	NS
SLC34A2	solute carrier family 34 (type II sodium/phosphate cotransporter), member 2b [Source:ZFIN;Acc:ZDB-GENE-030709-1]	4.6	2.6E−02	1.2	NS
SLCO2A1	solute carrier organic anion transporter family, member 2A1 [Source:ZFIN;Acc:ZDB-GENE-060606-3]	2.4	1.5E−03	1.2	NS
**DNA methylation**					
METTL21A	methyltransferase like 21A [Source:ZFIN;Acc:ZDB-GENE-050320-145]	4.4	2.0E−02	−1.3	NS
HNMT	histamine N-methyltransferase [Source:HGNC Symbol;Acc:HGNC:5028]	3.1	2.4E−03	1.2	NS
HMGCS1	3-hydroxy-3-methylglutaryl-CoA synthase 1 (soluble) [Source:ZFIN;Acc:ZDB-GENE-040426-1042]	2.8	1.0E−02	1.1	NS
TRMT11	tRNA methyltransferase 11 homolog (S. cerevisiae) [Source:ZFIN;Acc:ZDB-GENE-040426-953]	2.6	2.8E−02	−1	NS
METTL18	methyltransferase like 18 [Source:HGNC Symbol;Acc:HGNC:28793]	2.6	1.3E−02	−1.2	NS
TET3	tet methylcytosine dioxygenase 3 [Source:ZFIN;Acc:ZDB-GENE-060526-109]	−2.1	1.6E−02	−1.2	NS
METTL5	methyltransferase like 5 [Source:ZFIN;Acc:ZDB-GENE-041010-21]	−2.4	7.4E−03	1	NS
DPY30	dpy-30 histone methyltransferase complex regulatory subunit [Source:ZFIN;Acc:ZDB-GENE-040718-136]	−2.5	2.1E−03	−1.1	NS
TRMT44	tRNA methyltransferase 44 homolog (S. cerevisiae) [Source:ZFIN;Acc:ZDB-GENE-041010-189]	−3	8.2E−03	−1.2	NS
**Growth and development**					
IGFBP3	insulin-like growth factor binding protein 3 [Source:ZFIN;Acc:ZDB-GENE-040412-1]	4.9	1.9E–02	–1.4	NS
RXFP4	relaxin/insulin like family peptide receptor 4 [Source:HGNC Symbol;Acc:HGNC:14666]	2.7	3.1E–02	–	–
IGF2	insulin-like growth factor 2 [Source:RefSeq peptide;Acc:NP_001266572]	2.5	1.8E–02	1.1	NS
IGFBP7	insulin-like growth factor binding protein 7 [Source:ZFIN;Acc:ZDB-GENE-040426-2423]	1.8	3.0E–02	1	NS
IGF2R	insulin-like growth factor 2 receptor [Source:ZFIN;Acc:ZDB-GENE-041014-300]	–1.8	2.7E–02	–1.1	NS
THRB	thyroid hormone receptor beta [Source:ZFIN;Acc:ZDB-GENE-990415-268]	–2	2.5E–03	–	–
THRSP	thyroid hormone responsive [Source:ZFIN;Acc:ZDB-GENE-081022-19]	–4.8	1.3E–03	–	–
**Cell cycle machinery**					
CADM1	cell adhesion molecule 1a [Source:ZFIN;Acc:ZDB-GENE-080505-2]	8.2	1.3E–04	–1.3	NS
CEP152	centrosomal protein 152 [Source:ZFIN;Acc:ZDB-GENE-111005-1]	4.8	4.2E−04	1.2	NS
CEP57	centrosomal protein 57 [Source:HGNC Symbol;Acc:HGNC:30794]	2.9	2.6E−03	1.1	NS
CEP135	centrosomal protein 135 [Source:ZFIN;Acc:ZDB-GENE-041210-325]	2.3	3.4E−02	−1.1	NS
TACSTD2	epithelial cell adhesion molecule [Source:ZFIN;Acc:ZDB-GENE-040426-2209]	2.3	1.0E−03	1.2	NS
NCAM1	neural cell adhesion molecule 1a [Source:ZFIN;Acc:ZDB-GENE-990415-31]	−2.4	4.2E−02	−1.5	NS
CEBPW	centromere protein W [Source:ZFIN;Acc:ZDB-GENE-100922-200]	−3.9	4.4E−02	1.1	NS
CHL1	cell adhesion molecule L1-like b [Source:ZFIN;Acc:ZDB-GENE-091105-1]	−4.3	1.4E−03	1.1	NS
CENPF	centromere protein F [Source:ZFIN;Acc:ZDB-GENE-041111-205]	−5.6	7.0E−04	1.3	NS
NDC80	NDC80 kinetochore complex component [Source:ZFIN;Acc:ZDB-GENE-030131-904]	−5.9	3.0E−03	−1.0	NS
GOS2	G0/G1 Switch 2, Putative Lymphocyte G0/G1 Switch Gene	−8.2	7.5E−11	−43.4	1.6E−14
***Cyclin***					
CDKL5	cyclin dependent kinase like 5 [Source:HGNC Symbol;Acc:HGNC:11411]	2.6	8.0E−03	1.20	NS
CCNG1	cyclin G1 [Source:ZFIN;Acc:ZDB-GENE-020322-1]	2.2	3.8E−03	−1.00	NS
NUCKS1	nuclear casein kinase and cyclin-dependent kinase substrate 1a [Source:ZFIN;Acc:ZDB-GENE-040912-175]	2.0	9.9E−03	1.16	NS
MRRF	mitochondrial ribosome recycling factor [Source:ZFIN;Acc:ZDB-GENE-040704-12]	2.0	2.4E−02	−1.25	NS
CDK6	cyclin-dependent kinase 6 [Source:ZFIN;Acc:ZDB-GENE-060503-786]	1.9	2.0E−02	1.17	NS
CCNI	cyclin I [Source:ZFIN;Acc:ZDB-GENE-040426-2898]	1.7	3.0E−02	−1.15	NS
CCNT2	cyclin T2b [Source:ZFIN;Acc:ZDB-GENE-030131-183]	−1.6	4.1E−02	1.01	NS
CCNG2	cyclin G2 [Source:ZFIN;Acc:ZDB-GENE-021016-1]	−2.1	1.6E−02	−1.28	NS
CCNB2	cyclin B2 [Source:ZFIN;Acc:ZDB-GENE-030429-12]	−2.1	1.3E−02	−1.07	NS
CNNM1	cyclin and CBS domain divalent metal cation transport mediator 1 [Source:HGNC Symbol;Acc:HGNC:102]	−2.2	9.8E−03	1.05	NS
CDK1	cyclin-dependent kinase 1 [Source:ZFIN;Acc:ZDB-GENE-010320-1]	−3.2	9.3E−03	−1.16	NS
CCNB3	cyclin B3 [Source:ZFIN;Acc:ZDB-GENE-060929-684]	−12.9	4.5E−04	−1.04	NS
***Mitotic spindle dynamics***					
MZT2B	mitotic spindle organizing protein 2B [Source:ZFIN;Acc:ZDB-GENE-040801-87]	2.3	3.4E−02	−1.2	NS
CDK2	cyclin-dependent kinase 2 [Source:ZFIN;Acc:ZDB-GENE-040426-2741]	−2.4	4.8E−02	1.11	NS
NUSAP1	nucleolar and spindle associated protein 1 [Source:ZFIN;Acc:ZDB-GENE-030827-5]	−3.5	3.4E−03	−1.1	NS
BUB1	BUB1 mitotic checkpoint serine/threonine kinase [Source:ZFIN;Acc:ZDB-GENE-081104-75]	−4.5	6.1E−03	−1.0	NS
PLK1	polo-like kinase 1 (Drosophila) [Source:ZFIN;Acc:ZDB-GENE-021115-7]	−8.1	3.8E−04	−1.0	NS
**Apoptotic signals**					
VWA11	von Willebrand factor A domain containing 11 [Source:ZFIN;Acc:ZDB-GENE-141211-58]	44.3	1.01E−08	5	1.4E−02
ID4	inhibitor of DNA binding 4 [Source:ZFIN;Acc:ZDB-GENE-051113-208]	2.2	1.2E−03	−2.2	NS
TBRG4	transforming growth factor beta regulator 4 [Source:ZFIN;Acc:ZDB-GENE-091020-8]	1.9	4.4E−02	1.2	NS
TIGAR	tp53-induced glycolysis and apoptosis regulator a [Source:ZFIN;Acc:ZDB-GENE-060312-25]	1.6	3.2E−02	−1.4	NS
TIGAR	tp53-induced glycolysis and apoptosis regulator a [Source:ZFIN;Acc:ZDB-GENE-060312-25]	1.6	3.2E−02	−1.4	NS
RABGAP1	RAB GTPase activating protein 1 [Source:HGNC Symbol;Acc:HGNC:17155]	−1.7	2.9E−02	−1.1	NS
RAB6C	RAB6A, member RAS oncogene family [Source:ZFIN;Acc:ZDB-GENE-040426-2849]	−1.8	1.7E−02	1.1	NS
RAB4B-EGLN2	RAB4B, member RAS oncogene family [Source:HGNC Symbol;Acc:HGNC:9782]	−2.4	4.2E−02	1.4	NS
DDIT3	DNA-damage-inducible transcript 3 [Source:ZFIN;Acc:ZDB-GENE-070410-90]	−2.4	9.7E−03	−1.1	NS
RASGEF1B	RasGEF domain family member 1B [Source:HGNC Symbol;Acc:HGNC:24881]	−3.2	1.9E−03	−2.2	NS
RASL11A	RAS-like, family 11, member A [Source:ZFIN;Acc:ZDB-GENE-050417-384]	-4.5	2.0E−04	−1.5	NS
RAB29	RAB29, member RAS oncogene family [Source:HGNC Symbol;Acc:HGNC:9789]	−5.1	5.0E−03	−1.1	NS
DDIT4L	DNA damage inducible transcript 4 like [Source:HGNC Symbol;Acc:HGNC:30555]	−9.3	2.0E−04	−1.2	NS
**Others**					
KRT4	keratin 4 [Source:ZFIN;Acc:ZDB-GENE-000607-83]	7.1	2.10E−02	6	1.79E−02
ASTL	six-cysteine containing astacin protease 1 [Source:ZFIN;Acc:ZDB-GENE-070621-1]	6.1	8.30E−08	4.7	3.61E−04
RN7SKP275	RN7SKP275 (RNA, 7SK Small Nuclear Pseudogene 275) is a Pseudogene	6.1	1.30E−02	−2.3	2.78E−02
BPIFC	BPI Fold Containing Family C	4.6	1.00E−02	3.5	4.06E−02
ITI1H	inter-alpha-trypsin inhibitor heavy chain 1 [Source:ZFIN;Acc:ZDB-GENE-130530-650]	4	7.20E−06	2	3.16E−02
PENK	proenkephalin a [Source:ZFIN;Acc:ZDB-GENE-030729-31]	3.1	2.20E−02	−4.4	2.81E−02
PGLYRP2	peptidoglycan recognition protein 2 [Source:HGNC Symbol;Acc:HGNC:30013]	2.8	1.60E−03	4.6	1.74E−02
DIO2	Iodothyronine Deiodinase 2	2.8	0.003	1.2	NS
LECT2	leukocyte cell derived chemotaxin 2 [Source:HGNC Symbol;Acc:HGNC:6550]	2.5	7.00E−04	16.6	2.20E−06
APOA1	apolipoprotein A-Ia [Source:ZFIN;Acc:ZDB-GENE-990415-14]	1.7	3.40E−02	4.7	4.18E−03
APOH	apolipoprotein H [Source:HGNC Symbol;Acc:HGNC:616]	1.7	2.80E−02	3.3	1.53E−02
NCOA7	Nuclear Receptor Coactivator 7	−10.2	4.60E−15	−2.7	4.00E−02

**Table 3:** Representative list of GO terms significantly affected in the liver and testis of male tilapia exposed to BaP. Determined by Gene Set Enrichment Analysis (GSEA; *p < 0.05*, fold change ≥ 10%).

**Tissue**	**Gene set category**	**Name**	**Median fold change**	***p*-value**
**Liver**	**Biological process**	mitosis	−2.8	1.9E−04
		cell cycle	−2.5	2.7E−04
		triglyceride biosynthetic process	−3.0	7.9E−04
		long-chain fatty-acyl-CoA biosynthetic process	−3.2	8.2E−04
		mitotic cytokinesis	−6.6	1.1E−03
		cell-cell signaling	2.9	1.1E−03
		G2-M transition of mitotic cell cycle	−2.4	1.3E−03
		cytokinesis	−5.8	1.7E−03
		cellular response to calcium ion	−3.3	2.5E−03
		cellular response to organic substance	−3.9	3.3E−03
		synaptic transmission	−2.0	4.5E−03
		peptidyl-serine phosphorylation	−2.6	4.7E−03
		mitotic cell cycle	−2.4	7.3E−03
		activation of MAPK activity	−2.1	7.7E−03
		cell division	−2.4	1.1E−02
		lipid homeostasis	−5.0	1.3E−02
		exocytosis	−3.4	1.4E−02
		mitotic spindle assembly checkpoint	−4.5	1.5E−02
		response to ethanol	−2.3	1.8E−02
		cellular response to glucose stimulus	−2.0	2.0E−02
		negative regulation of signal transduction	2.1	2.0E−02
		DNA metabolic process	−4.2	2.0E−02
		fatty acid biosynthetic process	−2.7	2.1E−02
		microtubule-based movement	−3.2	2.4E−02
		cytokine-mediated signaling pathway	−2.2	2.4E−02
		epidermis development	2.1	2.4E−02
		positive regulation of MAPK cascade	2.5	2.5E−02
		sterol biosynthetic process	3.1	2.5E−02
		cellular lipid metabolic process	−2.5	2.7E−02
		positive regulation of JUN kinase activity	−3.9	3.0E−02
		apoptotic process	−2.1	3.1E−02
		cell surface receptor signaling pathway	1.8	3.2E−02
		response to testosterone	−2.5	3.2E−02
		lipid catabolic process	−3.2	3.2E−02
		actin cytoskeleton reorganization	−2.2	3.3E−02
		meiotic nuclear division	−3.4	3.9E−02
		positive regulation of gene expression	−2.1	3.9E−02
		immune system process	−1.7	4.4E−02
		biosynthetic process	2.9	4.6E−02
	**Cellular component**	spindle pole	−5.2	4.4E−05
		chromosome, centromeric region	−3.5	1.8E−04
		kinetochore	−4.5	3.5E−04
		proteinaceous extracellular matrix	−2.1	1.8E−03
		condensed chromosome kinetochore	−4.5	2.1E−03
		spindle	−2.8	2.8E−03
		chromosome	−2.4	3.3E−03
		spindle microtubule	−3.5	4.3E−03
		external side of plasma membrane	−1.9	4.5E−03
		extracellular region	−1.6	9.1E−03
		anchored component of membrane	−2.6	1.8E−02
		axon	−2.4	2.6E−02
		cell-cell junction	−1.7	2.7E−02
		cytoskeleton	−2.1	2.8E−02
		kinesin complex	−4.6	2.9E−02
		microtubule	−2.4	2.9E−02
		cell	−2.2	3.3E−02
		microtubule organizing center	−2.0	4.0E−02
	**Molecular function**	iron ion binding	−2.3	1.1E−03
		protein C-terminus binding	−2.7	1.1E−03
		microtubule binding	−2.7	3.2E−03
		heme binding	−1.9	4.2E−03
		protein heterodimerization activity	−2.4	5.8E−03
		protein serine-threonine kinase activity	−2.1	7.7E−03
		hormone activity	2.5	9.0E−03
		oxidoreductase activity, acting on paired donors, with incorporation or reduction of molecular oxygen	2.5	9.6E−03
		growth factor activity	2.5	1.2E−02
		drug binding	−3.3	1.2E−02
		metallopeptidase activity	−1.9	1.6E−02
		microtubule motor activity	−3.2	1.8E−02
		protein homodimerization activity	−2.2	2.8E−02
		calmodulin binding	−2.4	4.3E−02
		structural constituent of cytoskeleton	−2.7	4.7E−02
		carbohydrate binding	−2.0	4.8E−02
		sequence-specific DNA binding RNA polymerase II transcription factor activity	−2.4	4.9E−02
**Testis**	**Biological process**	regulation of proteolysis	9.3	9.4E−06
		proteolysis	10.0	7.1E−05
		leukotriene biosynthetic process	15.5	5.1E−04
		negative regulation of endopeptidase activity	9.3	9.6E−04
		hemostasis	10.3	2.3E−03
		oxygen transport	−7.5	5.9E−03
		protein heterooligomerization	−7.5	5.9E−03
		wound healing	7.5	7.3E−03
		fibrinolysis	6.0	1.6E−02
		cobalamin metabolic process	11.1	1.8E−02
		cellular protein metabolic process	8.6	3.0E−02
		response to calcium ion	5.5	3.7E−02
		inflammatory response	3.6	4.1E−02
		response to peptide hormone	9.3	4.4E−02
		response to cytokine	9.3	4.8E−02
	**Cellular component**	extracellular space	5.0	2.7E−06
		extracellular region	3.3	6.7E−05
		keratin filament	6.0	1.8E−04
		intermediate filament	6.0	3.7E−04
		hemoglobin complex	−7.5	4.1E−04
		Golgi lumen	−4.2	1.5E−03
		anchored component of external side of plasma membrane	15.5	5.6E−03
		secretory granule	8.6	2.7E−02
		platelet alpha granule	9.3	3.1E−02

**Table 4:** Partial list of subnetworks significantly affected in the liver and testis of male tilapia exposed to BaP (*p *<* *0.05, fold change ≥ 10%).

**Tissue**	**Gene set seed**	**Median fold change**	***p*-value**
**Liver**	kinetochore assembly	−3.2	2.5E−04
	telophase	−3.8	5.4E−04
	microtubule cytoskeleton assembly	−2.2	8.3E−04
	anaphase	−3.1	9.7E−04
	mitotic spindle positioning	−3.2	1.4E−03
	meiosis	−2.2	2.1E−03
	mitotic nuclear membrane assembly/disassembly	−3.2	2.3E−03
	microtubule/kinetochore interaction	−5.6	2.6E−03
	mitotic checkpoint	−3.2	3.0E−03
	mitotic spindle checkpoint	−3.2	3.2E−03
	nuclear division	−3.2	4.7E−03
	mitotic spindle assembly	−4.2	8.3E−03
	meiosis II	−4.5	8.8E−03
	fatty acid oxidation	−2.3	9.1E−03
	nuclear fragmentation	−2.3	9.7E−03
	sister chromatid cohesion	−3.2	1.0E−02
	chromosome condensation	−2.4	1.1E−02
	mitotic spindle orientation	2.4	1.1E−02
	spindle assembly	−2.7	1.4E−02
	Schwann cell migration	−3.2	1.5E−02
	DNA replication during S phase	−3.9	1.7E−02
	mitotic prometaphase	−3.2	1.7E−02
	mesenchymal stem cell differentiation	−1.9	1.8E−02
	synapse maturation	−2.4	2.0E−02
	monocyte differentiation	−2.5	2.0E−02
	mitotic sister chromatid segregation	−3.2	2.6E−02
	mitotic metaphase plate congression	−3.2	2.7E−02
	chromosome segregation	−2.8	2.8E−02
	regulation of action potential	1.7	2.9E−02
	adipogenesis	−2.2	3.0E−02
	nerve sprouting	−2.4	3.0E−02
	interphase	−2.4	3.0E−02
	microtubule bundling	−2.8	3.1E−02
	cellular extravasation	−3.1	3.1E−02
	lipid oxidation	−3.0	3.5E−02
	blood-retinal barrier	−2.0	3.7E−02
	Glycogen degradation	−2.2	3.8E−02
**Testis**	blood clotting	5.5	5.2E−04
	neutrophil chemotaxis	5.5	7.3E−04
	fibrinolysis	5.6	9.1E−04
	blood vessel permeability	3.7	1.2E−03
	myoblast proliferation	7.5	1.4E−03
	muscle fiber development	5.8	4.4E−03
	blood clot lysis	5.8	5.6E−03
	T-cell homeostasis	−2.3	5.7E−03
	neutrophil recruitment	3.6	7.6E−03
	degranulation	3.9	7.9E−03
	fibroblast proliferation	3.3	8.2E−03
	neutrophil adhesion	3.1	8.7E−03
	autolysis	7.5	1.1E−02
	zymogen activation	5.6	1.1E−02
	complement activation	5.0	1.2E−02
	hemolysis	3.0	1.5E−02
	chondrocyte proliferation	2.9	1.6E−02
	neutrophil extravasation	5.6	1.9E−02
	skin changes	7.5	1.9E−02
	blood coagulation, intrinsic pathway	5.8	2.0E−02
	cellular extravasation	3.6	2.1E−02
	hepatic regeneration	3.7	2.3E−02
	myoblast fusion	3.2	2.3E−02
	neutrophil migration	3.3	2.7E−02
	bacterial load	4.7	2.8E−02
	antigen expression	4.5	3.1E−02
	positive chemotaxis	3.6	3.2E−02
	superoxide anion generation	3.3	3.3E−02
	neutrophil activation	3.6	3.4E−02
	immune cell chemotaxis	3.6	3.5E−02
	hepatocyte apoptosis	5.8	3.7E−02
	dendritic cell differentiation	3.3	3.8E−02
	tissue invasion	5.0	3.8E−02
	muscle fiber contraction	3.0	3.9E−02
	myoblast differentiation	5.8	4.3E−02
	epidermal cell differentiation	6.0	4.4E−02
	innate immune response	3.3	4.4E−02
	leukocyte recruitment	3.1	4.7E−02

## 2 Experimental design, materials and methods

Liver and testis samples were collected as were controls. Analysis of gene expression profile RNA-Seq was performed as is mentioned in [1]. Briefly, Tilapia RNA-Seq reads were trimmed, clean reads aligned to reference genome using Tophat [3], [4]. Differential expression analysis was conducted using exact test with R package EdgeR (*p *< 0.05; fold change > ± 1.5 were considered as significant). Elsevier PathwayStudio^TM^ V9 (Elsevier, Inc., Rockville, MD, USA) operating with the ResNet 10.0 database was used to identify the biological mechanism that underlie the BaP effects. The gene set enrichment analysis (GSEA) and subnetwork enrichment analysis (SNEA) algorithms (applying the Mann−Whitney test with an alpha level of *p *<* *0.05) [5], [6], [7]. Quantitative PCR (qPCR) was used to evaluate the transcriptomic changes of key genes such as *Ddit4*, *Gadd45b* and *Igf2*, *Tet3* and *Fasn* involved in important functions, i.e, DNA damage, growth and development. The *rpl8* gene was used as the internal reference normalizer gene.

## References

[1] Colli−dula R.C. (2018). Transcriptome analysis reveals novel insights into the response of low−dose benzo(a)pyrene exposure in male tilapia. Aquat. Toxicol..

[2] Livak K.J., Schmittgen T.D. (2001). Analysis of relative gene expression data using real-time quantitative PCR and the 2−ΔΔCT method. Methods.

[3] Trapnell C. (2012). Differential gene and transcript expression analysis of RNA-seq experiments with TopHat and Cufflinks. Nat. Protoc..

[4] Trapnell C., Pachter L., Salzberg S.L. (2009). TopHat: discovering splice junctions with RNA-seq. Bioinformatics.

[5] Kotelnikova E. (2012). Novel approach to meta-analysis of microarray datasets reveals muscle remodeling-related drug targets and biomarkers in Duchenne muscular dystrophy. PLoS Comput. Biol..

[6] Sivachenko A., Kalinin A., Yuryev A. (2006). Pathway analysis for design of promiscuous drugs and selective drug mixtures. Curr. Drug Discov. Technol..

[7] Subramanian A. (2005). Gene set enrichment analysis: a knowledge-based approach for interpreting genome-wide expression profiles. Proc. Natl. Acad. Sci. USA.

