# Supplementary material for: Gene expression profile and molecular pathway datasets resulting from benzo(a)pyrene exposure in the liver and testis of adult tilapia
Source: Data Brief. 2018 Sep 5;20:1500–9. doi: 10.1016/j.dib.2018.08.206 (PMC6153355; doi:10.1016/j.dib.2018.08.206)

**Conflicts of Interest**

Authors have no conflicts of interest.

The corresponding author should sign this declaration on behalf of all the authors:


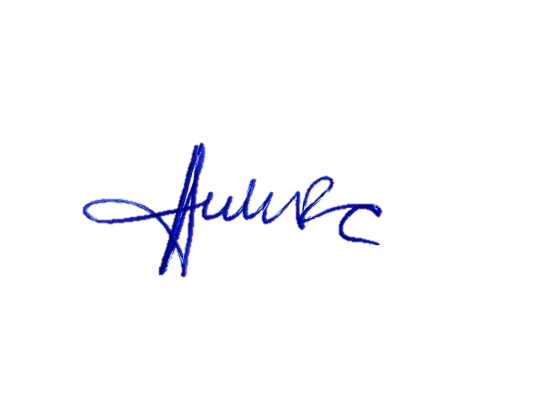

Supplement: Supplementary file 1 — Supplementary material [file mmc1.docx]
